# LncRNA SDCBP2-AS1 is a putative biomarker for postmenopausal osteoporosis and promotes osteogenic differentiation of BMSCs by regulating miR-361-3p

**DOI:** 10.1186/s41065-025-00494-5

**Published:** 2025-07-10

**Authors:** Jindong Chen, Shilong Zhang, Dixin Cai, Qing Yin, Qian Xie, Pengfang Xu, Junling Zhu

**Affiliations:** 1 Department of Orthopedics, The Affiliated Qingyuan Hospital (Qingyuan People’s Hospital), Guangzhou Medical University, Qingyuan, 511518 China; 2 Department of Orthopedics and Traumatology, Shanghai Fengxian District Hospital of Traditional Chinese Medicine, Shanghai, 201499 China; 3 Department of Orthopedics, The First Affiliated Hospital of Xiamen University, Xiamen, 361001 China; 4 Chongqing Dianjiang County Hospital of Traditional Chinese Medicine, Dianjiang, 408399 China; 5 Department of Orthopedics, The People’s Hospital of Dazu, Chongqing, Dazu, 402360 China; 6 Department of Traditional Chinese Medicine, Ningbo No.2 Hospital, No. 41 West North Street, Haishu District,, Ningbo, 315099 Zhejiang China

**Keywords:** Postmenopausal, Osteoporosis, BMSC, SDCBP2-AS1, miR-361-3p, Diagnosis

## Abstract

**Background:**

Numerous long noncoding RNAs (lncRNAs) have been proven to participate in osteogenesis and postmenopausal osteoporosis (PMOP). We measured serum SDCBP2-AS1 expression changes in patients with PMOP and investigated its effects on osteoblast differentiation in human bone marrow-derived mesenchymal stem cells (hBMSC) cells.

**Methods:**

RT-qPCR was used to measure SDCBP2-AS1 levels and the expression of osteogenic differentiation indicators. The diagnostic efficacy of SDCBP2-AS1 was assessed using a receiver operating characteristic (ROC) analysis. CCK-8 and flow cytometry methods were employed to investigate the functional impact of SDCBP2-AS1 on hBMSC cell proliferation and apoptosis during osteoblast differentiation. The bioinformatics, dual-luciferase reporter assay, and RNA Immunoprecipitation (RIP) assay were used to identify and confirm SDCBP2-AS1/miR-361-3p interaction.

**Results:**

Serum SDCBP2-AS1 was decreased in patients with PMOP, especially in those with fractures. The SDCBP2-AS1 levels were positively correlated with patients’ T scores and BMDs. Decreased SDCBP2-AS1 had a certain high area under the ROC curve (AUC) value (AUC = 0.81) in distinguishing PMOP patients with fractures from those without fractures. SDCBP2-AS1 levels gradually increased after four weeks of treatment in PMOP patients and hBMSCs during cell differentiation. Enhanced SDCBP2-AS1 promoted cell proliferation and the levels of osteoblast differentiation markers, including ALP, OCN, RUNX2, and Collagen I, while decreasing cell apoptosis. miR-361-3p was a direct target of SDCBP2-AS1. The influence of SDCBP2-AS1 on cell activities and hBMSCs differentiation was diminished by miR-361-3p.

**Conclusions:**

SDCBP2-AS1 might be a diagnostic biomarker in predicting PMOP patients with fractures. By measuring the levels of SDCBP2-AS1 in patient samples, clinicians may be able to identify those who are more susceptible to bone fractures, enabling earlier and more targeted preventive measures. SDCBP2-AS1 targeting miR-361-3p regulates the osteogenic differentiation of hBMSCs, which might be a new target for the treatment of PMOP.

**Supplementary Information:**

The online version contains supplementary material available at 10.1186/s41065-025-00494-5.

## Background

Postmenopausal osteoporosis (PMOP) is a systemic bone metabolic disease that occurs when women enter menopause, leading to decreased secretion of estrogen, bone loss and bone microstructure degradation, resulting in increased bone fragility and increased risk of fracture [1, 2]. At present, the main treatment for osteoporosis is drug therapy [3, 4] and there are other non-pharmacological therapies for osteoporosis treatment [5]. Although there are many kinds of drugs, in terms of the final curative effect on patients, most of them only improve the clinical symptoms and delay the further development of the diseases, and do not achieve the effect of reversing the diseases or even curing [6–8]. Human bone marrow-derived mesenchymal stem cells (hBMSCs) are mesenchymal stem cells that abundant in bone marrow and cancellous bone. hBMSCs have osteogenic differentiation potential and are the seed cells of osteoblasts, which play a crucial regulatory role in maintaining bone homeostasis [9, 10]. However, many molecules and signaling pathways involved in the osteogenic differentiation of BMSCs are still unknown [11], and extensive research is needed to uncover the relevant mechanisms and effectively induce osteogenesis in BMSCs, to provide new directions for the treatment of osteoporosis.

Increasing studies demonstrated that non-coding RNAs (ncRNAs), especially long non-coding RNAs (lncRNAs) and microRNAs (miRNAs), have crucial biological functions, which have great research value as potential biomarkers and therapeutic targets for diagnosis, prognosis, and treatment [12, 13]. Increasing evidence confirmed the crucial role of ncRNAs in musculoskeletal conditions, such as tendon injuries [14, 15], osteoarthritis [16], osteoporosis [17], and rheumatoid arthritis [18]. lncRNAs (> 200 nt) are functional RNAs and participate in various pathological processes by regulating miRNAs or downstream genes in numerous diseases, including PMOP [19]. For instance, lncRNA KCNQ1OT1 mitigates PMOP by enhancing the proliferation, migration, and osteogenic differentiation of MC3T3-E1 cells by regulating miR-421-3p/mTOR pathway [20]. LncRNA syndecan-binding protein 2‐antisense RNA 1 (SDCBP2-AS1), located on Chromosome 20, was involved in some diseases, such as ovarian cancer and Alzheimer’s disease [21, 22]. A previous silico analysis study indicated that SDCBP2-AS1 was one of the abnormally expressed lncRNAs in osteoporotic patients [23]. However, whether SDCBP2-AS1 has clinical performance and functional effects in PMOP remains unclear.

Many studies show miRNAs modulate the differentiation of osteoblasts and osteoclasts, thereby exerting a significant impact on the process of bone remodeling [24, 25]. miRNAs could be regulated by lncRNAs to modulate disease development by promoting or inhibiting cellular phenotype of cells [26, 27]. The aberrant expression of miR-361-3p was observed in many diseases and participated in the disease progression [28–30]. For instance, miR-361-3p overexpression could inhibit cementoblast differentiation [31]. A study focused on miRNA-gene interaction networks using GEO datasets pointed miR-361-3p was an upregulated miRNA in osteoporosis cases [32]. However, the role of miR-361-3p in PMOP and whether has functional effects on hBMSC cells remain elusive.

Nevertheless, there remains a vacancy in the comprehensive understanding of the roles of SDCBP2-AS1 and miR-361-3p in PMOP. Our research aimed to investigate the clinical influence of SDCBP2-AS1 in PMOP and figure out whether the regulatory link existed between SDCBP2-AS1 and miR-361-3p in PMOP. The functional influence of SDCBP2-AS1/miR-361-3p in hBMSC was also explored.

## Methods

### Study subjects and specimen preparation

A total of 162 female patients with PMOP (64.17 ± 5.41 years) who were admitted to The First Affiliated Hospital of Xiamen University were enrolled in this study from February 2022 to December 2023. According to the presence or absence of fragility fracture, they were divided into a fracture group and a non-fracture group, including 78 OF patients and 84 simple PMOP patients. The included criteria: (1) postmenopausal women over 55 years, (2) Natural menopause time > 1 year, (3) met the diagnostic criteria for osteoporosis, (4) all patients had not received any previous anti-osteoporosis treatment. Besides, according to matched age, body mass index (BMI), and menopausal age, healthy postmenopausal women (*n* = 88) with normal BMD who underwent physical examination were enrolled as the control group. The control individuals had no history of previous fractures, especially fractures related to osteoporosis. None of the enrolled individuals had a history of medications known to influence bone metabolism (e.g., hormonal therapies, antiepileptic drugs, diuretics, and calcium supplements). The age, age at menopause, BMI, T score, and 25(OH)D were collected and recorded. The BMD examination results calculated by Dual-emission X-ray Absorptiometry system (Lunar Prodigy) were collected, including BMD at hips, femoral neck, and the first through the fourth lumbar vertebrae. The fasting venous blood was collected from each participant. The specimens were centrifuged at 1500 g for 10 min to separate the supernatant serum. Then the serum specimens were stored in -80 °C freezer until use.

The procedures used in this study adhere to the tenets of the Declaration of Helsinki. All individuals involved in the study were advised of its objectives and provided signed consent forms. The research protocol received ethical clearance from the Ethics Committee at The First Affiliated Hospital of Xiamen University.

### Cell culture and osteogenic differentiation stimulation

hBMSCs were procured from the ATCC (PCS-500-012, Manassas, VA, USA) at passages 3–5 and cultured in DMEM (Gibco, USA) supplementary with 10% FBS (Invitrogen, Carlsbad, USA) at 37 °C in a humidified condition of 5% CO_2_.

For the induction of osteoblastic differentiation, the hBMSCs were incubated in a specialized osteogenic differentiation medium for 14 days, which comprised 200 µM ascorbic acid, 10 mM β-glycerophosphate, and 100 nM dexamethasone (Sigma-Aldrich, USA). The culture medium was refreshed every three days.

### Cell transfection

SDCBP2-AS1 siRNA (si-SDCBP2-AS1), siRNA negative control (si-NC), pcDNA3.1-SDCBP2-AS1 (oe-SDCBP2-AS1), pcDNA3.1 vector (oe-NC), miR-361-3p mimic, and mimic NC were obtained from GenePharma (Shanghai, China). Sequences are shown in Supplementary Table 1. For transfection, all the oligonucleotides were transfected into hBMSCs cells using Lipofectamine 2000 (Invitrogen). The cells were incubated with the transfection reagent for 4–6 h and the serum-free DMEM medium was replaced with a complete DMEM medium. After 48 h of incubation, subsequent analyses were performed.

### Total RNA isolation and RT-qPCR

With the help of RNAzol RT RNA Isolation Reagent (iGeneBio, Guangdong, China), total RNA was isolated from serum samples. After detecting the quality and quantity of total RNA, reverse transcription was performed utilizing a High-Capacity cDNA Reverse Transcription Kit (ThermoFisher, USA) for lncRNA and mRNAs, or TaqMan^®^ MicroRNA Reverse Transcription Kit for miRNA [33, 34]. PCR amplification was conducted with the SYBR Green qPCR Master Mix (ThermoFisher) on an ABI 7500HT real-time PCR system. Sequences are shown in Supplementary Table 1. To calculate the relative quantities of lncRNA, mRNA, and miRNA, the 2^−ΔΔCt^ method was performed after normalization with endogenous reference GAPDH and U6, respectively.

### RNA Immunoprecipitation (RIP) assay

The RIP assay was performed using the Magna RIP™ RNA-Binding Protein Immunoprecipitation Kit from Millipore. Briefly, 500 µg of total RNA from cell lysate was incubated with beads conjugated to either 5 µg of anti-Ago2 or control anti-IgG for 2 h. The immunocomplexes were subsequently eluted and subjected to treatment with DNase and Proteinase K to remove DNA and proteins, respectively. Post-purification, the precipitated RNAs were extracted and reverse transcribed. Subsequent quantification of the target transcripts was performed via qPCR analysis.

### Osteogenic differentiation marker detection

To substantiate the osteogenic function of SDCBP2-AS1, the osteogenic differentiation markers, including ALP, OCN, RUNX2, and Collagen I, were detected using RT-qPCR analysis. The related sequences were as follows: Forward, 5’-TGTGCGGGGTCAAGGCTAAC-3’; reverse, 5’-GGCGTCCGAGTACCAGTTGC-3’; OCN, forward 5’-CCTGAGTCTGACAAAGCCTTC-3’ and reverse 5’-GCGGTCTTCAAGCCATACTG-3’; RUNX2, forward: 5’-CGTCCACTGTCACTTTAATAGCTC-3’, and reverse: 5’-GTAGCCAGGTTCAACGATCTG-3’; and Collagen I, forward: 5’-CTGGCGGTTCAGGTCCAAT-3’; reverse: 5’-TCCAAACCACTGAAGCCTCG-3’.

### Cell counting kit 8 (CCK-8) assay

Cells (2000 cells/well) were seeded in 96-well plates. The viability of cells was detected by CCK-8 kit (Dojindo) at 24, 48, and 72 h after inoculation. Briefly, 10 µL CCK-8 solutions were introduced to each well, followed by a 1-hour of incubation period. The absorbance at 450 nm was measured using a microplate spectrophotometer.

### Cell apoptosis assay

The apoptosis abilities were assessed by flow cytometry (Becton Dickinson, San Diego, CA, USA) using Annexin V-FITC Apoptosis Detection Kit (BD Biosciences). After the transfection, cells were washed with PBS and resuspended in a binding buffer, followed by incubation with V-FITC and PI in the dark. Followed by FCM (BD FACS Calibur, BD, USA) using the FL1 channel for Annexin V-FITC and the FL2 channel of PI. Both early apoptotic (Annexin V+/PI-) and late apoptotic (Annexin V+/PI+) cells are included in the cell assay of apoptosis. Then, apoptotic cells were analyzed by Flow J software (BD Biosciences).

### Dual-luciferase reporter assay

The downstream miR-361-3p of SDCBP2-AS1 was predicted by StarBase V2.0 database (https://rnasysu.com/encori/index.php) (last update: 10/18/2024) [35]. The 3’UTR fragments of SDCBP2-AS1 that contained wild-type (WT) or mutant (MUT) binding sites for miR-361-3p were amplified and cloned into pGLO-Report luciferase vector (Promega, Shanghai, China) to construct the SDCBP2-AS1-Wt or SDCBP2-AS1-Mut plasmids, respectively. Then these plasmids were co-transfected with miR-361-3p mimic or mimic NC utilizing Lipofectamine 2000 for 48 h. The luciferase reporter activities were measured by a dual-luciferase assay kit.

### Statistical analysis

All the experiments were repeated at least three times and the data were presented with mean ± SD. The data were analyzed utilizing GraphPad Prism 9.0 (San Diego, CA, USA). Continuous variables were compared using Student’s t-test (for two groups) or one-way ANOVA (for multiple groups), with post-hoc Tukey’s test for pairwise comparisons. Correlations between SDCBP2-AS1 and clinical parameters were assessed via Pearson’s correlation coefficient. For diagnostic performance, the receiver operating curve (ROC) was conducted to evaluate the diagnostic value of SDCBP2-AS1 using the GraphPad software with the calculation of the area under the curve (AUC) [36, 37]. Results were deemed statistically significant when the *p*-value fell below 0.05.

## Results

### Demographic characteristics data

The basic demographic characteristics and BMD data are shown in Table 1. The age, age at menopause, and BMI did not differ significantly between PMOP patients and controls (*P* > 0.05). The T score, total hip BMD, femoral neck BMD, L1-4 BMD, and 25(OH)D were lower in PMOP patients than in controls.

**Table 1:** Demographic characteristics of PMOP patients and health control

Variable	Study group		*P*-value
	Control	PMOP	
Age (year)	63.77 ± 4.98	64.17 ± 5.41	0.57
Age at menopause (year)	49.91 ± 3.33	50.29 ± 2.96	0.35
BMI (kg/m^2^)	23.76 ± 2.29	24.00 ± 2.42	0.46
T score	-0.68 ± 0.98	-3.16 ± 0.45	< 0.001
Total hip BMD (g/cm^2^)	1.05 ± 0.09	0.70 ± 0.06	< 0.001
Femoral neck BMD (g/cm^2^)	1.06 ± 0.11	0.67 ± 0.08	< 0.001
L1-4 BMD (g/cm^2^)	1.06 ± 0.12	0.66 ± 0.07	< 0.001
25(OH)D (ng/mL)	34.95 ± 6.37	26.34 ± 5.05	< 0.001

### SDCBP2-AS1 expression in patients with PMOP and its correlation with BMD levels

**Fig. 1:**
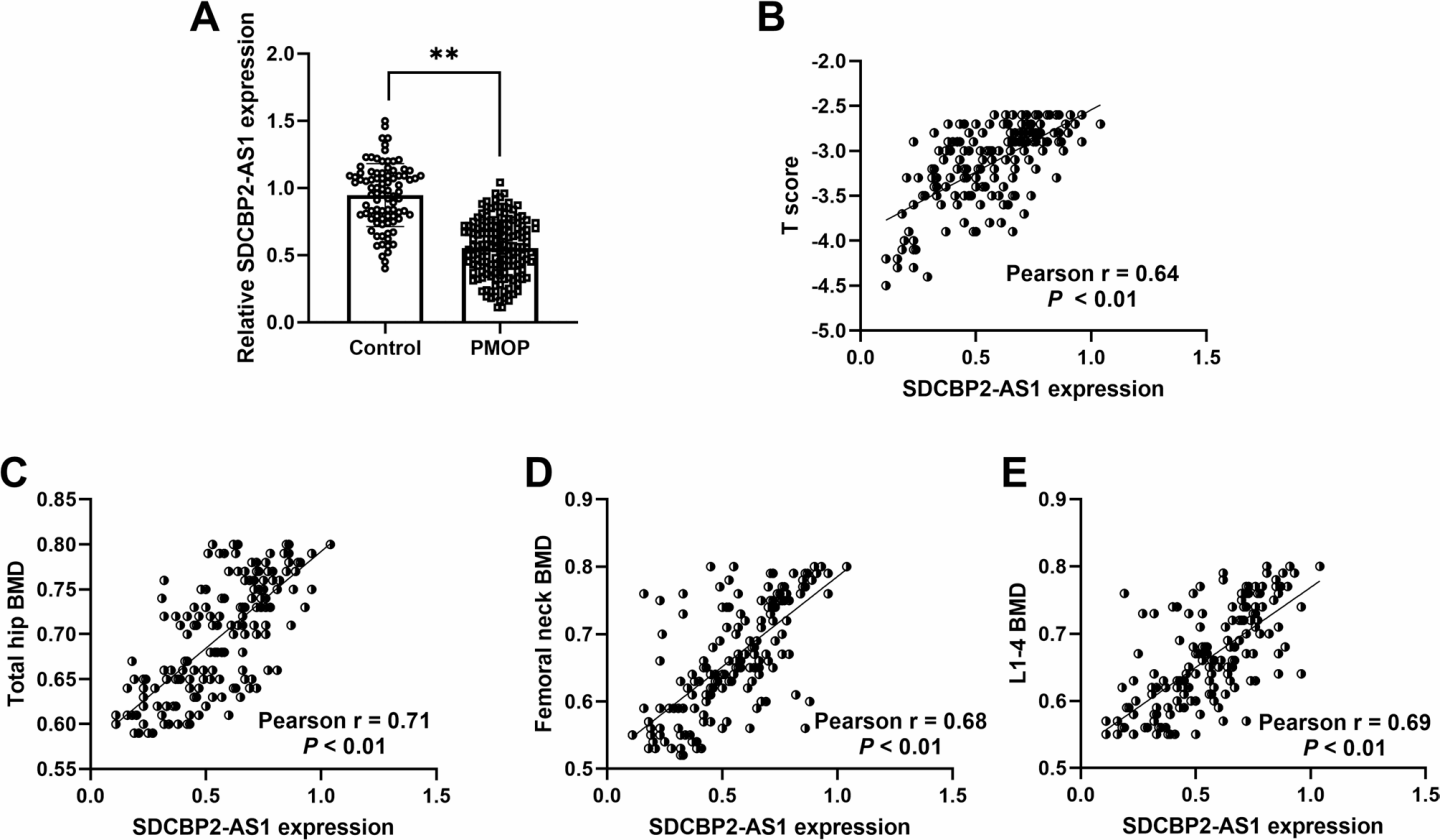
lncRNA SDCBP2-AS1 expression in patients with PMOP. (**A**) Serum SDCBP2-AS1 was downregulated in patients with PMOP compared to controls. (**B**) There is a positive correlation between SDCBP2-AS1 expression and T scores in patients with PMOP. (**C-E**). A positive relationship was observed between SDCBP2-AS1 expression and total hip BMD, femoral neck BMD, and L1-4 BMD levels. **P < 0.01

Student t-test showed that patients with PMOP exhibited lower SDCBP2-AS1 relative expression in contrast to controls (Fig. 1A). Moreover, SDCBP2-AS1 levels were positively correlated with T score (Pearson *r* = 0.64, *P* < 0.01, Fig. 1B), total hip BMD (*r* = 0.71, *P* < 0.01, Fig. 1C), femoral neck BMD (*r* = 0.68, *P* < 0.01, Fig. 1D), and L1-4 BMD (*r* = 0.69, *P* < 0.01, Fig. 1E).

### SDCBP2-AS1 expression in PMOP patients with and without fracture

**Fig. 2:**
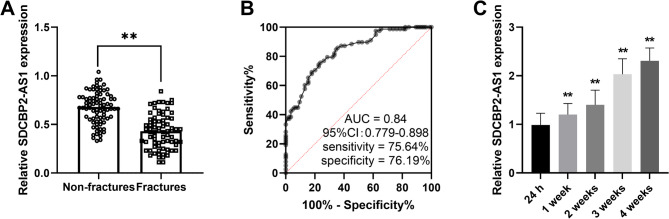
The expression and clinical role of SDCBP2-AS1 in PMOP patients with fractures. (**A**) Serum SDCBP2-AS1 expression was lower in PMOP patients with fractures than in patients without fractures. (**B**) ROC curve indicated that SDCBP2-AS1 has potential diagnostic value in distinguishing patients with fractures from those without fractures with a relatively high AUC value. (**C**) SDCBP2-AS1 levels were gradually increased after treatment in patients with fractures. **P < 0.01

In PMOP patients, 78 patients had fractures, and 84 patients had no fractures (simple PMOP). SDCBP2-AS1 expression was lower in patients with fractures than in PMOP patients without fractures (Fig. 2A). The area under the ROC curve of 0.84 (95%CI = 0.779–0.898) showed a good diagnostic value in distinguishing patients with fractures from those without fractures with a sensitivity of 75.64% and specificity of 76.19% (Fig. 2B). The PMOP patients with fractures received surgery and anti-osteoporotic treatment and the SDCBP2-AS1 levels increased (Fig. 2C).

### The influence of SDCBP2-AS1 on osteoblast activity in hBMSCs

**Fig. 3:**
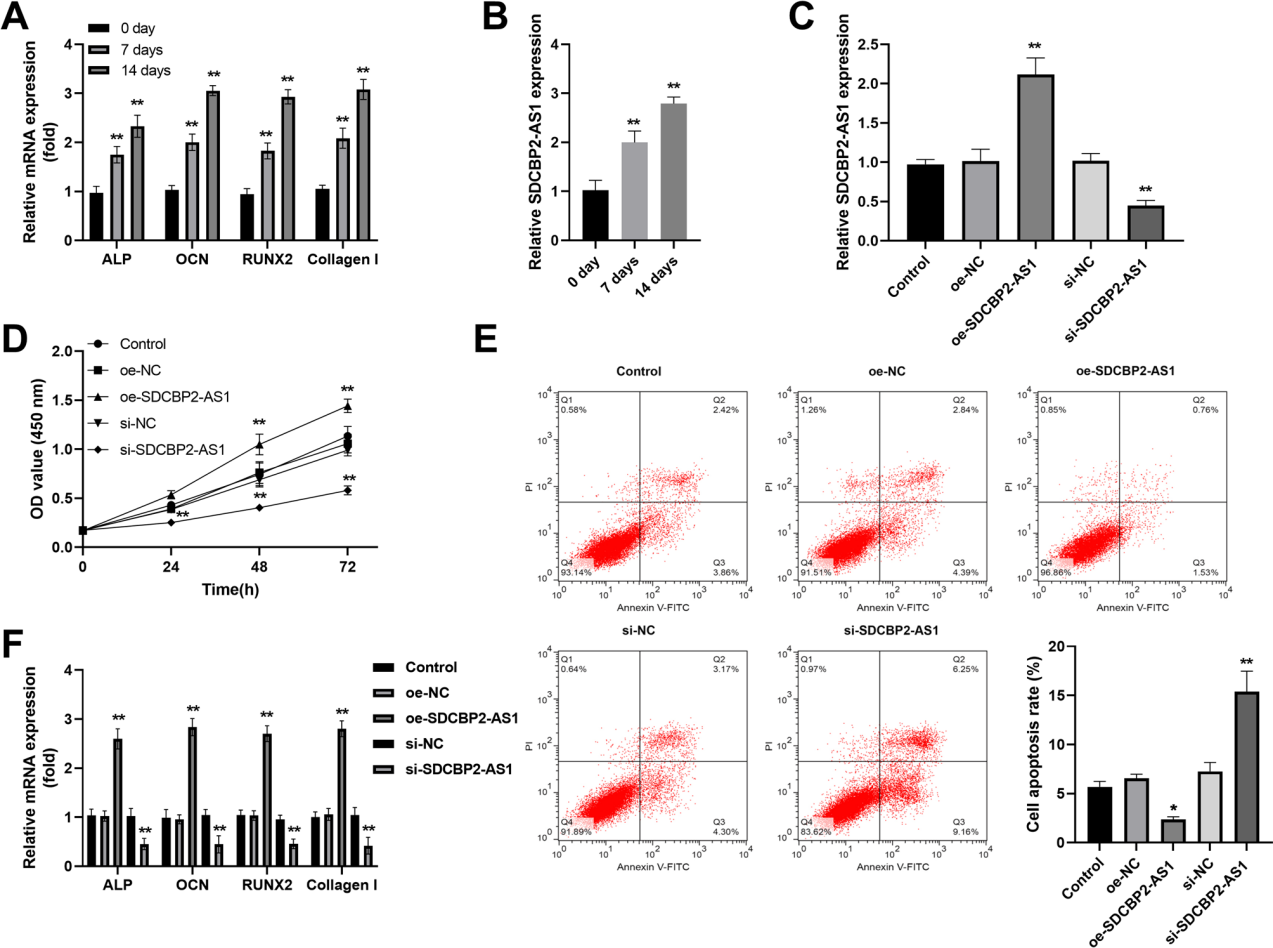
The effect of SDCBP2-AS1 on osteoblast activity in hBMSCs. (**A**) The osteogenic differentiation indicators ALP, OCN, RUNX2, and Collagen I mRNA levels increased in osteoblast differentiation. (**B**) SDCBP2-AS1 expression gradually increased during osteoblast differentiation. (**C**) RT-qPCR method was utilized to detect the transfection efficiency in hBMSCs. (**D**) Overexpression of SDCBP2-AS1 promoted cell proliferation, while knockdown of its expression inhibited cell proliferation. (**E**) Enhanced SDCBP2-AS1 repressed cell apoptosis, while decreased expression of SDCBP2-AS1 promoted cell apoptosis. (**F**) The levels of ALP, OCN, RUNX2, and Collagen I increased in oe-SDCBP2-AS1 transfected cells, while decreasing in si-SDCBP2-AS1 transfected cells. **P < 0.01

With the osteogenic differentiation time, the osteogenic differentiation indicators ALP, OCN, RUNX2, and Collagen I mRNA levels were increased in hBMSCs (Fig. 3A). Then the expression of SDCBP2-AS1 was detected during osteogenic stimulation (0,7, 14 days) and the levels of SDCBP2-AS1 also showed an increasing trend in hBMSCs (Fig. 3B). To explore the effects of SDCBP2-AS1 on osteogenic activities in hBMSCs, transfection was carried out. The results indicated that oe-SDCBP2-AS1 promoted the SDCBP2-AS1 expression and si-SDCBP2-AS1 repressed its expression in hBMSC cells (Fig. 3C). CCK-8 assay demonstrated that upregulation of SDCBP2-AS1 promoted hBMSC cell proliferation, whereas depletion of SDCBP2-AS1 inhibited hBMSC cell proliferation during osteogenic differentiation (Fig. 3D). On the contrary, overexpression of SDCBP2-AS1 inhibited cell apoptosis while silencing SDCBP2-AS1 promoted cell apoptosis in hBMSC cells (Fig. 3E). Consistent with the trend on cell proliferation, the influence of SDCBP2-AS1 on osteogenic differentiation markers (ALP, OCN, RUNX2, and Collagen I) was observed (Fig. 3F).

### miR-361-3p was a direct target of SDCBP2-AS1

**Fig. 4:**
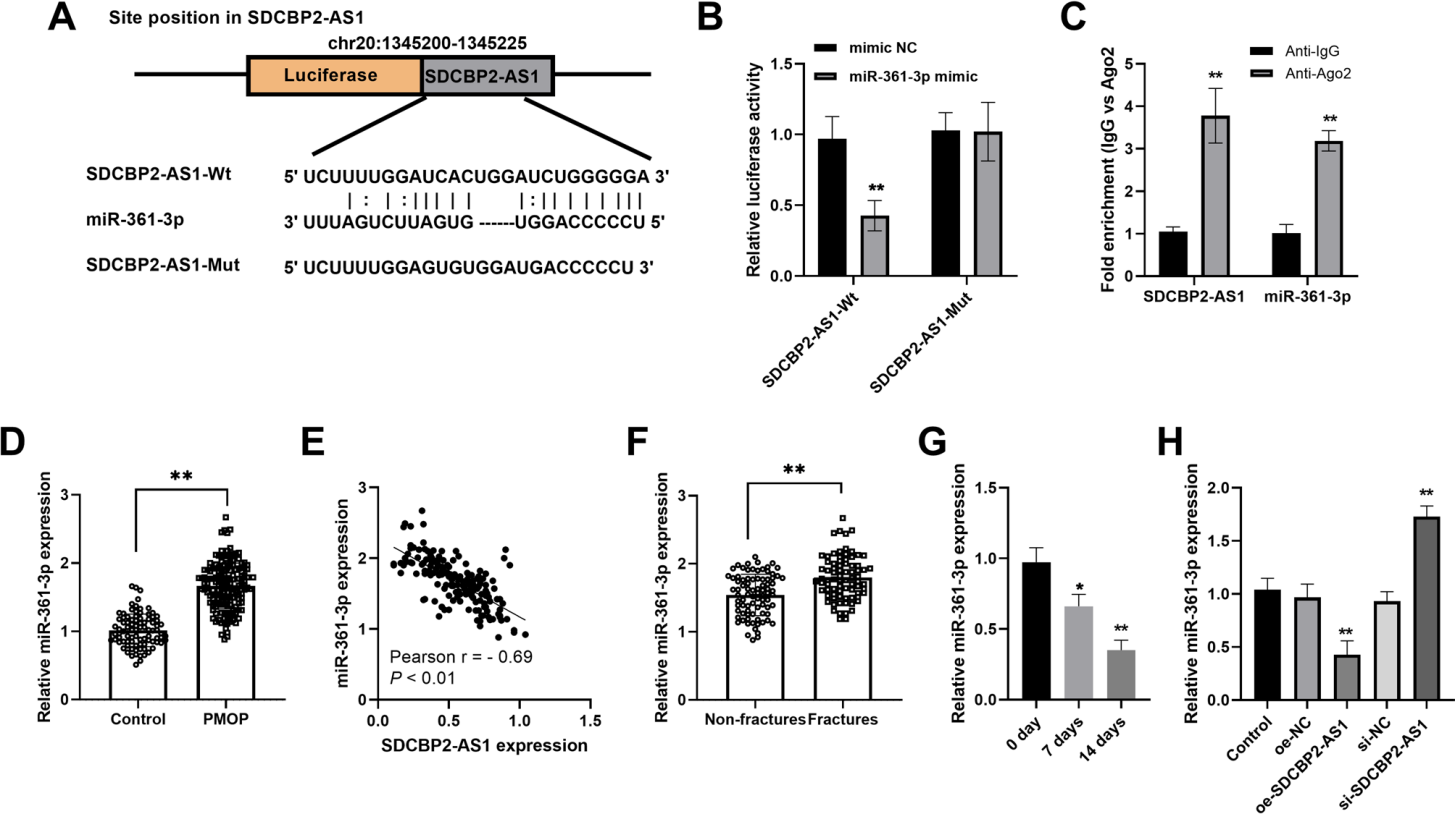
miR-361-3p was a direct target miRNA of SDCBP2-AS1. (**A**) The StarBase database predicted the binding sites between SDCBP2-AS1 and miR-361-3p. (**B**) Dual-luciferase reporter assay confirmed the binding relationship between SDCBP2-AS1 and miR-361-3p. (**C**) RIP assay further confirmed both SDCBP2-AS1 and miR-361-3p were enriched on anti-Ago2. (**D**) Serum miR-361-3p was upregulated in patients with PMOP. (**E**) A negative correlation between SDCBP2-AS1 and miR-361-3p was observed in patients with PMOP. (**F**) An increased expression miR-361-3p was observed in patients with fractures. (**G**) miR-361-3p gradually decreased during osteoblast differentiation. (**H**) miR-361-3p expression was decreased in oe-SDCBP2-AS1 transfected cells while increased in si-SDCBP2-AS1 transfected cells. *P < 0.05, **P < 0.01

The StarBase database predicted and displayed the binding sites between SDCBP2-AS1 and miR-361-3p (Fig. 4A). The dual-luciferase reporter assay indicated that elevated expression of miR-361-3p inhibited the luciferase activity of SDCBP2-AS1-Wt vector (Fig. 4B). RIP assay indicated that both SDCBP2-AS1 and miR-361-3p were enriched in AGO2 antibody compared to IgG (Fig. 4C). miR-361-3p was upregulated in patients with PMOP compared to controls (fold change 1.67, *P* < 0.01, Fig. 4D) and displayed a negative correlation with SDCBP2-AS1 expression (Fig. 4E). An increased miR-361-3p expression was observed in PMOP patients with fractures (Fig. 4F). During osteogenic differentiation stimulation, a downtrend expression of miR-361-3p was observed (Fig. 4G). Besides, miR-361-3p was downregulated in SDCBP2-AS1-upregulated cells, while was upregulated in SDCBP2-AS1-silencing cells (Fig. 4H).

### miR-361-3p partially reversed the effects of SDCBP2-AS1 on osteoblast activities in hBMSCs

**Fig. 5:**
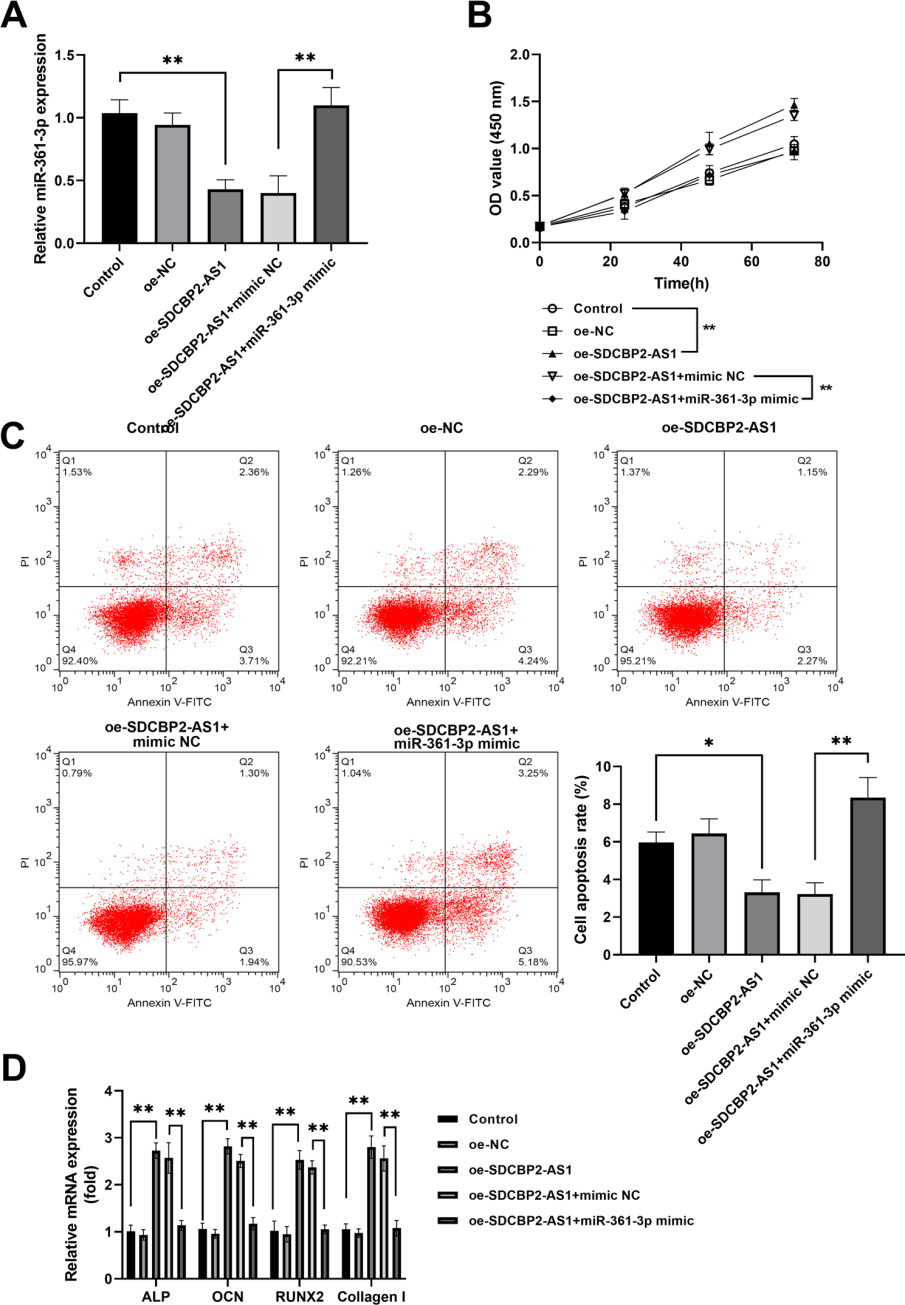
miR-361-3p partially reversed the effects of SDCBP2-AS1 on osteoblast activities in hBMSCs. (**A**) RT-qPCR was utilized to detect the miR-361-3p expression after co-transfection. (**B**) Overexpression partially reversed the enhanced proliferation abilities caused by oe-SDCBP2-AS1. (**C**) The inhibited apoptosis by SDCBP2-AS1 was reversed by enhanced miR-361-3p expression. (**D**) Elevation of osteoblast differentiation-related markers (ALP, OCN, RUNX2, and Collagen I) expression by SDCBP2-AS1 was partially reversed by miR-361-3p overexpression. *P < 0.05

After co-transfection, miR-361-3p expression was decreased in SDCBP2-AS1-upregulated hBMSCs cells, while the decreased expression was attenuated after co-transfection of miR-361-3p mimic (Fig. 5A). CCK-8 assay indicated that miR-361-3p overexpression abolished the promotion of SDCBP2-AS1 on cell proliferation (Fig. 5B). Flow cytometry apoptosis analysis demonstrated that the inhibited apoptosis by SDCBP2-AS1 was reversed by enhanced miR-361-3p expression (Fig. 5C). Moreover, the elevation of osteoblast differentiation-related markers (ALP, OCN, RUNX2, and Collagen I) by SDCBP2-AS1 was partially reversed by miR-361-3p overexpression (Fig. 5D).

## Discussion

In the current study, SDCBP2-AS1 expression in serum was reduced in PMOP patients compared with age-matched healthy controls and positively associated with T scores and BMD. Decreased SDCBP2-AS1 expression was correlated with an increased likelihood of fractures and it exhibited a potential diagnostic value in differentiating PMOP patients with fractures from those without fractures. During osteogenic differentiation stimulation, SDCBP2-AS1 expression gradually increased with induction time and could regulate osteogenic activities and osteoblast differentiation by targeting miR-361-3p.

More and more researchers are trying to explore biomarkers as a therapy to monitor osteoporosis [38–40]. lncRNAs exist widely in many organisms and participate in various diseases [41, 42]. An increased number of studies have reported that the abnormal expression of lncRNAs is involved in bone development, formation, and bone-related diseases [43–45]. In this study, serum SDCBP2-AS1 was downregulated in patients with PMOP and especially in PMOP patients with fractures. The data was consistent with the results of SDCBP2-AS1 in the GEO dataset [32]. The decreased SDCBP2-AS1 expression was positively correlated with low BMP levels in patients with PMOP. These data suggest that SDCBP2-AS1 might play a candidate role in PMOP. The identification of promising biomarkers, being of great significance in appraising the initial diagnosis and the efficacy of treatments, is of utmost critical priority. Patients with PMOP are prone to the occurrence of fractures. However, both the PMOP patients with and without fractures have low T scores and BMPs, thus, timely identification of patients with PMOP who are at risk of fractures is extremely important. Based on the lower SDCBP2-AS1 levels in patients with fractures, the ROC curve showed that SDCBP2-AS1 expression has a relatively high AUC value in distinguishing PMOP patients with fractures from those without fractures. After treatment, SDCBP2-AS1 levels increased in PMOP patients with fractures. These data suggest that SDCBP2-AS1 might be a potential diagnostic biomarker in predicting the risk of fractures in PMOP patients.

The aberrant expression of SDCBP2-AS1 was reported in other diseases, wherein it exerts its influence by modulating cellular activities. For instance, SDCBP2-AS1 was upregulated in lung cancer cells and depletion of its expression could promote lung cancer cell ferroptosis by regulating miR-656-3p/CRIM1 axis [46]. Conversely, SDCBP2-AS1 is downregulated in gastric cancer tissues and suppresses cell proliferation and migration of gastric cancer cells by causing SUMOylation of hnRNP K and stabilized β-catenin activity [47]. Bone formation is a crucial phase in the healing process of fracture. The influence of SDCBP2-AS1 on osteoblasts was further explored. During the osteogenic induction using hBMSCs, the levels of osteogenic differentiation markers ALP, OCN, RUNX2, and Collagen I gradually increased, as well as SDCBP2-AS1 expression also gradually increased. This data indicated that SDCBP2-AS1 might have a functional role in fracture repair. Overexpression of SDCBP2-AS1 facilitated hBMSC cell proliferation, osteogenic differentiation markers expression, and inhibited cell apoptosis. Knockdown of SDCBP2-AS1 inhibited hBMSC cell proliferation, osteogenic differentiation markers expression, and promoted cell apoptosis during osteogenic differentiation. Our in vitro data show SDCBP2-AS1 promoted osteoblast proliferation and differentiation, which confirmed the crucial role of SDCBP2-AS1 during fracture healing.

miR-361-3p was an elevated miRNA in patients with osteoporosis and might involve focal adhesion, PI3K-Akt signaling pathway, and Gap junction [32]. miR-361-3p was also upregulated in osteosarcoma and facilitated tumorigenesis of osteosarcoma cells through regulating ARID3A [48]. The findings in this study demonstrated that miR-361-3p could be directly bound to SDCBP2-AS1 and miR-361-3p expression was elevated in patients with PMOP. Functionally, overexpression of miR-361-3p partially mitigated the effects of SDCBP2-AS1 on hBMSC cell activities and the process of osteoblast differentiation. Collectively, these results indicated that miR-361-3p served as a direct target of SDCBP2-AS1 in the underlying mechanism.

However, the current study still has several limitations. Firstly, the expression of SDCBP2-AS1 was measured in serum samples, which lack of animal or patient tissue validation beyond serum levels. This study explored the effects of SDCBP2-AS1 on cell apoptosis, but did not explore the changes in cell cycle phase. Thus, further research on the cell cycle in future studies will be conducted. Moreover, the current study focused mainly on in vitro cell-based experiments, and the translation of these findings to the in vivo context of PMOP patients remains to be explored. Primary human cells and animal models of PMOP should be utilized to better understand the role of SDCBP2-AS1 in PMOP. In addition, whether SDCBP2-AS1 plays a crucial role in PMOP by regulating miR-361-3p through the PI3K-Akt signaling pathway remains unclear. To clarify this, future studies will employ pathway-specific inhibitors to explore the detailed mechanism of SDCBP2-AS1 in PMOP and PMOP-caused fracture. In future, the function of SDCBP2-AS1/miR-361-3p in POMP will be explored in an animal fracture model or clinical sample panel, which may provide targets for the traditional medicine for diseases [49, 50].

In conclusion, SDCBP2-AS1 downregulation is associated with PMOP and fragility fracture risk, serving as a promising diagnostic biomarker. Enhanced expression of SDCBP2-AS1 might promote osteogenic differentiation through regulating miR-361-3p in the underlying mechanism, which might be treatment targets of PMOP. The present study provided a certain theoretical basis for further studies on the correlation between lncRNA and PMOP-caused fractures.

## Electronic supplementary material

Below is the link to the electronic supplementary material.

Supplementary Material 1

## Data Availability

The datasets used and/or analysed during the current study are available from the corresponding author on reasonable request.
