# Supplementary material for: LncRNA SDCBP2-AS1 is a putative biomarker for postmenopausal osteoporosis and promotes osteogenic differentiation of BMSCs by regulating miR-361-3p
Source: Hereditas. 2025 Jul 10;162:124. doi: 10.1186/s41065-025-00494-5 (PMC12247319; doi:10.1186/s41065-025-00494-5)
Supplement: Supplementary file 1 — Supplementary Material 1 [file 41065_2025_494_MOESM1_ESM.docx]

Supplementary Table 1 Sequences used in the study

|  | Sequences 5'-3' |
| --- | --- |
| si-SDCBP2-AS1 | 5'-TACATTATTATTAACTATAGTCT-3' |
| si-NC | 5'-UUCUCCGAACGUGUCACGUTT-3' |
| miR-361-3p mimic | 5'-UCCCCCAGGUGUGAUUCUGAUUU-3' |
| mimic NC | 5'-UUCUCCGAACGUGUCACGUTT-3' |
| SDCBP2-AS1 |  |
| forward | 5'-GCAGGGCCCTTTGAACCTAT-3' |
| reverse | 5'-TGGAAGCCACCAAGATGTCC-3' |
| miR-361-5p |  |
| forward | 5'-GCCGAGUCCCCCAGGUGUGAU-3' |
| reverse | 5'-CTCAACTGGTGTCGTGGA-3' |
| GAPDH |  |
| forward | 5'-CCTGGAGAAACCTGCCAAGT-3' |
| reverse | 5'-TAGCCCAGGATGCCCTTTAG-3' |
| U6 |  |
| forward | 5'-GCTTCGGCAGCACATATACTAAAAT-3' |
| reverse | 5'-CGCTTCACGAATTTGCGTGTCAT-3' |
